# The effects of historical fragmentation on major histocompatibility complex class II β and microsatellite variation in the Aegean island reptile, *Podarcis erhardii*

**DOI:** 10.1002/ece3.3022

**Published:** 2017-05-18

**Authors:** Trent Santonastaso, Jackie Lighten, Cock van Oosterhout, Kenneth L. Jones, Johannes Foufopoulos, Nicola M. Anthony

**Affiliations:** ^1^ Department of Biological SciencesUniversity of New OrleansNew OrleansLAUSA; ^2^ School of Environmental SciencesUniversity of East AngliaNorwich Research ParkNorwichUK; ^3^ Department of Biochemistry and Molecular GeneticsUniversity of Colorado Denver School of MedicineDenverCOUSA; ^4^ School of the Environment and SustainabilityUniversity of MichiganAnn ArborMIUSA

**Keywords:** drift, historical fragmentation, immunity, major histocompatibility complex, selection

## Abstract

The major histocompatibility complex (MHC) plays a key role in disease resistance and is the most polymorphic gene region in vertebrates. Although habitat fragmentation is predicted to lead to a loss in MHC variation through drift, the impact of other evolutionary forces may counter this effect. Here we assess the impact of selection, drift, migration, and recombination on MHC class II and microsatellite variability in 14 island populations of the Aegean wall lizard *Podarcis erhardii*. Lizards were sampled from islands within the Cyclades (Greece) formed by rising sea levels as the last glacial maximum approximately 20,000 before present. Bathymetric data were used to determine the area and age of each island, allowing us to infer the corresponding magnitude and timing of genetic bottlenecks associated with island formation. Both MHC and microsatellite variation were positively associated with island area, supporting the hypothesis that drift governs neutral and adaptive variation in this system. However, MHC but not microsatellite variability declined significantly with island age. This discrepancy is likely due to the fact that microsatellites attain mutation‐drift equilibrium more rapidly than MHC. Although we detected signals of balancing selection, recombination and migration, the effects of these evolutionary processes appeared negligible relative to drift. This study demonstrates how land bridge islands can provide novel insights into the impact of historical fragmentation on genetic diversity as well as help disentangle the effects of different evolutionary forces on neutral and adaptive diversity.

## 1 INTRODUCTION

Understanding how fragmentation affects genetic variation and viability of wild populations is of primary importance to conservation biology. Small isolated populations in fragmented systems can lose genetic variation and diverge rapidly from one another through genetic drift (Frankham, 2003; Wright, 1931). This loss of variation can lead to decreased fitness (Bouzat, 2010; Gos, Slote, & Wright, 2012; Miller, Allendorf, & Daugherty, 2010), either through inbreeding depression or loss of adaptive evolutionary potential (Charlesworth & Charlesworth, 1987; Frankham, 1995; Saccheri, Brakefield, & Nichols, 1996). Fitness may also be affected in small populations through a reduction in functional diversity at genes involved in critical immunological processes. Rising extinction rates due to emerging pathogens and habitat loss therefore make it particularly important to understand how population fragmentation affects potentially adaptive genetic diversity at key immunity loci. (Altizer, Harvell, & Friedle, 2003).

An important set of genes involved in the adaptive immune response is the major histocompatibility complex (MHC). The MHC is the most polymorphic region in the vertebrate genome (Bjorkman & Parham, 1990) and has provided significant insight into the impact of parasite‐mediated selection on host genetic variation in natural populations (Bernatchez & Landry, 2003; Sommer, 2005). MHC molecules are present on the surface of host cells and are responsible for presenting foreign peptides to helper T‐cells thereby mediating an adaptive immune response (Murphy, Travers, & Walport, 2008). The MHC Class I proteins are present on the surface of all vertebrate nucleated somatic cells and facilitate immune responses to intracellular parasites (Bjorkman & Parham, 1990) whereas class II proteins are present on specific antigen‐presenting cells and combat extracellular pathogens (Kappes & Strominger, 1988).

The high level of variability observed in the MHC is often attributed to one or more forms of parasite‐mediated selection, namely: overdominance (heterozygote advantage; Sommer, 2005), negative frequency‐dependent selection (rare‐allele advantage; Bernatchez & Landry, 2003) or fluctuating selection (reviewed in Bernatchez & Landry, 2003; Sommer, 2005; Spurgin & Richardson, 2010). At local levels, parasites could reduce genetic variation at the MHC through selection for specific disease‐resistant alleles, resulting in a form of short‐term local adaptation (Eizaguirre, Lenz, Kalbe, & Milinski, 2012; Kyle et al., 2014). However, at greater spatial and temporal scales, these patterns of selection may shift, ultimately increasing genetic diversity relative to that of neutral markers (Castro‐Prieto, Wachter, & Sommer, 2011). Studies have also shown that gene conversion within the MHC can generate haplotype diversity and recover functional variation in bottlenecked populations (Spurgin et al., 2011). Ultimately, the relative impact of selection and drift on MHC variation will depend not only on the intensity and mode of selection but also on levels of immigration, recombination, and demographic history (Chen, Bei, & Li, 2015; Eimes et al., 2011; Spurgin et al., 2011; Wilson, Whittington, & Bahr, 2014). Unfortunately, these factors are rarely examined in studies of MHC in natural populations and can only be properly addressed with sufficient replication and a detailed knowledge of the duration and intensity of the population bottleneck under investigation (Oliver & Piertney, 2012).

Islands in the Cyclades group of the central Aegean represent an ideal study system for examining the effects of historical bottleneck events on MHC diversity. While the Aegean Sea constitutes a complex evolutionary laboratory (Lymberakis & Poulakakis, 2010), the situation in the Cycladic archipelago is more straightforward and is characterized by remarkable geologic stability (Lykousis, 2009). Since the most recent glacial maximum (GLM) 20,000 years before present (BP), global sea levels have risen 120–130 m across much of the Aegean Sea, fragmenting a once contiguous landmass into a series of land bridge islands (Kapsimalis et al., 2009; Lykousis, 2009; Sakellariou & Galanidou, 2016). These continental shelf islands were historically connected to one another during the GLM and have been progressively fragmented since that time by rising sea levels (Kapsimalis et al., 2009). Bathymetric data have shown that islands can be grouped into clusters that reflect a common fragmentation history, that is, each fragmentation cluster contains islands that were more recently separated from each other than islands in other fragmentation clusters (Foufopoulos & Ives, 1999; Kapsimalis et al., 2009). Regional bathymetric data have also provided precise estimates of island area and age (Foufopoulos & Ives, 1999), allowing us to infer the corresponding magnitude and timing of each fragmentation event.

**Figure 1:**
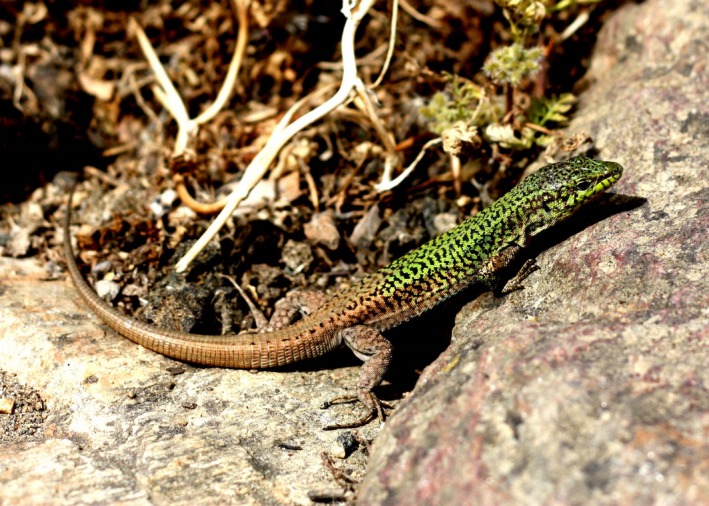
*Podarcis erhardii* in typical habitat on Naxos, of the Cyclades in the Aegean Sea

The Aegean wall lizard *Podarcis erhardii* (Figure 1) is an ideal study organism for assessing the effects of island fragmentation on adaptive variation. With few exceptions, this species is found throughout Cyclades and does not readily disperse between islands (Hurston et al., 2009; Roca, Foufopoulos, Valakos, & Pafilis, 2009). In keeping with the bathymetric data, hierarchical analyses of molecular variance have shown that mitochondrial cytochrome *b* variation is largely structured by island fragmentation cluster (Hurston et al., 2009). Studies of nuclear microsatellite loci have also shown that neutral variation is positively associated with island area and weakly negatively associated with island age, consistent with the cumulative effects of drift (Hurston et al., 2009). However, the effects of fragmentation history on adaptive (MHC) genetic variation have not yet been assessed.

This study therefore capitalizes on this unique continental shelf island system to assess how drift, selection, migration, and recombination have impacted MHC variability in *P. erhardii* populations that have been subject to fragmentation events of different magnitude (island area) and duration (island age). If balancing selection is the most important evolutionary force in this study system, MHC variation should persist regardless of the magnitude or age of the fragmentation event. If migration is present, MHC differentiation should be less than that of neutral markers because the efficacy of gene flow is higher for loci under balancing selection (see McMullan & van Oosterhout, 2012; Muirhead, 2001; Schierup, Vekemans, & Charlesworth, 2000). Conversely, if migration is negligible, we expect MHC differentiation to exceed that of neutral markers reflecting local adaptation in response to divergent selection. If our hypothesis of balancing selection is not supported and drift is the dominant evolutionary force we expect that MHC differentiation will reflect those observed at neutral loci. Under this scenario, neutral and MHC variation will increase with island area and decrease with island age. Under a model where drift predominates but migration is occurring between islands, we expect to find a pattern of isolation by distance or little to no differentiation between islands. Lastly, we examine the extent of recombination within the MHC as this process can act to counter the loss of genetic variation in severely fragmented populations.

## 2 MATERIALS AND METHODS

### 2.1 Sampling

**Figure 2:**
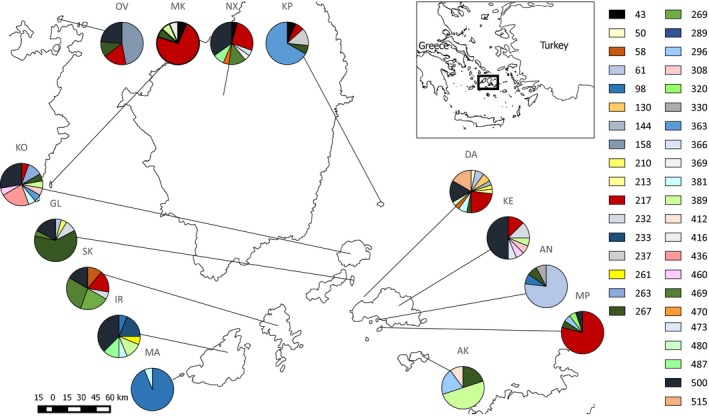
Map of central Cyclades in the Aegean Sea. Pie charts are clustered by fragmentation group and represent the frequency of different MHC class II β alleles in each population as indicated by the relevant colors). Population codes are as follows: AK—Antikeros, AN—Andreas, DA—Daskalio, GL—Glaronissi, IR—Irakleia, KE—Keros, KO—Koufonissi, KP—Kopria, MA—Megalos Ambelas, MK—Makronissi, MP—Megali Plaka, NX—Naxos, OV—Ovriokastro, SK—Schoinoussa. Numbers refer to individual Poer‐DAB* alleles

Three hundred *P. erhardii* lizards were sampled from 15 islands within the Cyclades region of the Aegean Sea, Greece (Figure 2). These islands range in size and age, and can be clustered into four groups (Amorgos, Naxos, Keros and Iraklia; Table 1) based on their shared fragmentation history (Foufopoulos & Ives, 1999). All islands are dominated by a characteristic sparse, spinose dwarf bush steppe (“phrygana”) and exposed rock glades with the exception of Naxos which harbors a higher diversity of more mesic vegetation types. Lowlands on Naxos tend to be used for agriculture while more temperate sites in the highlands contain copses, olive groves, and maquis. *Podarcis erhardii* inhabits all of these habitats with the exception of small closed‐canopy forest habitats. On small islands, (<10 ha) lizards were sampled opportunistically from across the island, whereas on larger islands (>10 ha) lizards were sampled at one site, in order to avoid the potentially confounding effects of population structure. Lizards were captured using a combination of silk noose and mealworm baits. Tissue was collected from toe or tail clips and stored in 95% ethanol. Genomic DNA was extracted using phenol–chloroform, as described in Sambrook, Russell, and Maniatis (2001).

**Table 1:** Characteristics of the Aegean islands included in this study

Island	Island group^a^	Island code^b^	N (MHC)^c^	N (microsatellite)^d^	Size (ha)^e^	Age (Kya)^f^	Distance (Km)^g^
Antikeros	Amorgos	AK	10	19	164	13.5	6.88
Andreas	Keros	AN	13	17	5	8.55	0.05
Daskalio	Keros	DA	19	30	1.5	1.50	0.10
Keros	Keros	KE	13	13	1,505	9.15	8.93
Megali Plaka	Keros	MP	17	25	3	6.45	0.40
Glaronissi	Irakleia	GL	19	26	18.75	5.65	0.55
Irakleia	Irakleia	IR	13	17	1807.8	9.80	5.36
Koufonissi	Irakleia	KO	15	16	1300	8.35	4.46
Megalos Ambelas	Irakleia	MA	16	24	7	7.85	0.35
Schoinoussa	Irakleia	SK	16	21	883	6.25	5.35
Kopria	Naxos	KP	13	27	13.75	11.7	4.20
Makronissi	Naxos	MK	12	24	4.2	5.85	1.05
Naxos	Naxos	NX	17	30	44,800	0	0
Ovriokastro	Naxos	OV	15	25	22	5.75	0.63

a Island group refers to the final fragmentation group to which each island belongs.

b Abbreviations of each island used throughout the text.

c Number of individuals used in analyses of the major histocompatibility complex.

d Number of individuals used in microsatellite analyses.

e Island area reported in hectares.

f Island age refers to how many thousands of years ago the land bridge to the nearest largest land mass submerged.

g Distance refers to the distance of each island to the nearest largest land mass.

### 2.2 Development of species‐specific MHC class II primers

A 539‐base pair (bp) fragment spanning exons 2 and 3 of the MHC class II β – 1 subunit was amplified from reverse‐transcribed cDNA of a single *P. erhardii* sample using degenerate primers MHC2Ex2F2 and MHC2Ex3R2 (Miller, Belov, & Daugherty, 2005; Supplementary Methods). Species‐specific forward and reverse primers (PodEx2F1 5′‐TCCTGTTCCAGTCGAAGGG‐3′ and PodEx2R1 5′‐ CCGGGACTGRGMGAAGGG‐3′) were then designed from these cDNA sequences to amplify a 225‐bp fragment of exon 2 encompassing the presumed peptide‐binding region (PBR).

### 2.3 Analysis of MHC variation

MHC class II sequencing was carried out using a 454 sequencing approach similar to that described by Babik, Taberlet, Ejsmond, and Radwan (2009). Sequences with incomplete barcodes, imperfect primer matches, or those not of the expected length (i.e., 223 ± 3 bp) were first removed from the dataset. Sequences with indels that did not differ by multiples of three were also discarded as they would result in frameshifts in the amino acid sequence. We then genotyped individuals based on the Degree of Change (DOC) in cumulative read depth within each amplicon to estimate the number of alleles per individual (*A*_i_; Lighten, van Oosterhout, Paterson, McMullan, & Bentzen, 2014). For a more detailed explanation of this method, see Lighten, van Oosterhout, Paterson, et al. (2014), Lighten, van Oosterhout, and Bentzen (2014) and Supplementary Methods.

We used *A*_i_ as a simple proxy for individual heterozygosity as described in Miller et al. (2010), because we were unable to assign MHC alleles to specific loci when using co‐amplifying primers. Allelic richness (AR_MHC_) was calculated through a rarefaction algorithm implemented in CONTRIB v.1.02 (Petit, el Mousadik, & Pons, 1998). The number of private alleles restricted to one population (P) was also recorded. ARLEQUIN v.3.1 (Excoffier, Laval, & Schneider, 2005) was used to estimate the theta (θ) parameter based on the average number of nucleotide differences between sites (θ_π_) or the number of segregating sites (θ_k_) within each population. Pairwise F_ST_ values were calculated in ARLEQUIN by entering the number of individuals with a given allele in each population as haplotype data (following Miller et al., 2010). A Mantel test was used to test for isolation by distance where Euclidian distances between islands were measured in Google EARTH v.7.0.3.8542 using the geographic center of each island as a reference point. An Analysis of Molecular Variance (AMOVA) was also carried out in ARLEQUIN to assess the percentage of overall variance attributable to among‐fragmentation groups and among‐ and‐within island populations, respectively. Pie charts depicting the frequencies of each MHC allele were mapped using Quantum GIS 2.4.0 (QGIS Development Team http://qgis.osgeo.org). Signatures of selection and/or demographic change were assessed in each population using the Tajima's *D* (Tajima, 1989) and Fu's F (Fu & Li, 1993) statistic as implemented in ARLEQUIN. The significance of these statistics was calculated using 1,000 coalescent simulations of the data. We also assessed the significance of Tajima's *D* within each population using a sliding window approach implemented in the program DNAsp (Librado & Rozas, 2009) using a three‐base pair window and a three‐base pair step. This sliding window approach was also used to identify‐specific residues displaying significant Tajima's *D* values. A Student's *t*‐test was used to assess whether there were significant differences in levels of MHC variability between islands exhibiting a signature of balancing selection relative to those that did not. Finally, we searched for evidence of microrecombination between alleles using the methods implemented in the RDP 3.44 platform (Martin et al., 2010). Aligned sequences were scanned for evidence of recombination using a 30‐base pair window and a step size of three for the following analyses: RDP, BOOTSCAN, SISCAN, PHYLPRO, TOPAL, and a window of ten segregating sites for MAXCHI and CHIMAERA. For GENECOV, only sequence triplets were scanned. Effective population size of each island population was estimated using the three single‐sample methods implemented in NeESTIMATOR (Waples, Peel, Macbeth, Tillet, & Ovenden, 2014).

### 2.4 Comparison of mitochondrial, microsatellite, and MHC variability

To compare estimates of MHC variability with those of neutral markers, we used the mitochondrial and microsatellite dataset of Hurston et al. (2009). This dataset is based on a 447‐base fragment of the cytochrome *b* gene and five microsatellite loci sampled from the same 15 island lizard populations examined in the present study. Microsatellite allelic richness was calculated using the rarefaction method implemented in HPRAR v.1.1 (Kalinowski, 2005). Pairwise F_ST_ values were similarly estimated for microsatellite data using ARLEQUIN and a Mantel test was conducted to test for isolation by distance. AMOVA analyses were carried out on mitochondrial and microsatellite data in order to provide a neutral estimate of population structure.

To quantify the amount of contemporary gene flow between islands, we estimated the proportion of contemporary migrants between populations using a Bayesian approach implemented in the program BAYESASS v 1.3 (Wilson & Rannala, 2003). The MCMC chain was run for a length of 2 × 10^6^ steps, a sampling interval of a 2,000 steps, and the first 25% of samples were discarded as burn‐in. We set the migration mixing parameter to 0.1, the allelic frequency mixing parameter to 0.4 and the inbreeding coefficient mixing parameter to 0.75, leading to an acceptance rate of approximately 0.3 which is well within the recommended range (Wilson & Rannala, 2003).

### 2.5 Effect of island area, age, and isolation on microsatellite and MHC variation

Bivariate and multiple regression analyses were carried out to assess the relationship between island area, age, isolation and their interactions with one another on microsatellite, and MHC diversity. Generally, age denotes the duration of the population bottleneck that each island lizard population has experienced. In most cases, this simply reflects the duration of an island's separation from its ancestral landmass. However, Naxos represents the largest remaining land fragment (>20 times larger than the next smallest island) with populations of *P. erhardii* likely numbering in the hundreds of thousands. It is hence extremely unlikely that *P. erhardii* could have experienced a population bottleneck on this island, and it was therefore given an age of zero. Measures of microsatellite diversity comprise: average expected heterozygosity (*H*_e_) and allelic richness (AR_msat_). For MHC data these comprise *A*_i_, AR_MHC_ and molecular diversity estimates θ_π_ and θ_k_ and P.

Prior to model testing, we carried out a Spearman rank correlation test between island variables in order to assess whether any of them were significantly correlated with one another. As distance was significantly correlated with the square‐root of island age (ρ = 0.594, *p* < .05) we removed it from all models to avoid effects of collinearity. We also mean‐centered the interaction term between age and area because of the significant correlation between log area*Age and age (ρ = 9096.4, *p* < .001). The interaction term was important to include as the effects of island area may be contingent on island age. Of the explanatory variables examined, only island area was not normally distributed and was therefore log transformed (Vittinghoff, Glidden, Shiboski, & Mcculloch, 2011). We tested for linearity by plotting the residuals of the regression analysis against their predicted values. We tested for evidence of significant outliers in the regression analyses using Cook's Distance measure (Cook, 1977). For each response variable, we conducted multiple regression analysis on saturated models, and removed explanatory variables and their interaction terms one at a time until the best fitted model was determined via the Akaike Information Criteria (AIC). Findings from these regression models were compared to a best subset regression analysis. As each explanatory variable was used in multiple models, we also used the Holm (Holm, 1979) correction for multiple hypothesis testing. Unless otherwise noted, all statistical analyses were conducted in R v.2.13.1 (R Core Development Team, 2015).

### 2.6 MHC phylogeny

MHC class II alleles were aligned using the MUSCLE algorithm in MEGA 6 (Tamura, Dudley, Nei, & Kumar, 2007). The substitution model that best fit the data was determined using J‐MODEL TEST 2.1.3 (Darriba, Taboada, Doallo, & Posada, 2012). Gene trees were estimated using maximum likelihood (ML) performed in ClustalX (Thompson, Gibson, Plewniak, Jeanmougin, & Higgins, 1997). The Bayesian analysis was run in MrBayes v.3.2.1 (Ronquist & Huelsenbeck, 2003) using Metropolis‐coupled Markov chain Monte Carlo (MCMC) sampling and an GTR + Γ + I substitution model as determined in J‐MODEL TEST (Darriba et al., 2012).

## 3 RESULTS

### 3.1 Patterns of MHC variability

Thirteen distinct MHC class II sequences of 539 bp in length were amplified from *P. erhardii* cDNA using previously described degenerate primers (Miller et al., 2005). Basic Local Alignment Search Tool (BLAST; Altschul, Gish, Miller, Myers, & Lipman, 1990) surveys of the NCBI database showed that all of these sequences had the highest nucleotide and amino acid identity to other reptile MHC class II ‐ β sequences. As predicted, all sequences span exons 2 and 3 of the reptilian MHC class II ‐ β gene. None of the translated sequences revealed any evidence of frameshifts or stop codons (Fig. S1).

Of the 300 individuals amplified using the species‐specific PodEx2F1/R1 MID‐primer combination, 89 samples failed to amplify and were subsequently dropped from sequence analysis. Although 15 islands were included in the original sampling design and 454 MHC sequencing, only three individuals from Agrilou (AG) amplified. As a result, we dropped this island from further analyses, leaving a total of 208 individuals sampled from 14 island populations for MHC analyses. Following 454 sequencing, sequence variants with incomplete barcodes were removed, yielding 538 sequence variants. After applying the DOC approach, 39 MHC alleles were retained. All of these alleles were 223 bp in length and translated into 37 unique amino acid sequences (Fig. S2). *A*_i_ did not show any significant association with the number of reads per amplicon (Fig. S3; range = 1–1,501, average = 265), indicating that coverage within amplicons was of sufficient depth to adequately estimate the number of alleles per amplicon.

**Figure 3:**
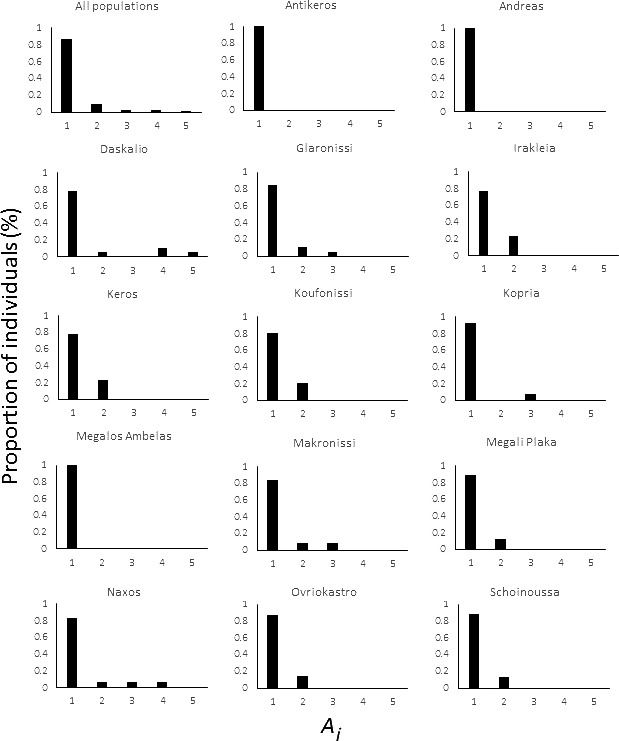
Frequency distributions of alleles per individual (*A*_i_) estimates for each island population of *Podarcis erhardii*

Across populations, average *A*_i_ ranged from 1.00 to 1.58, and AR_MHC_ from 0.31 to 3.12 (Table 2). Of the 208 genotyped individuals, 180 (86.5%) possessed one allele, 20 (9.6%) possessed two alleles, four (1.9%) possessed three alleles, three (1.4%) possessed four alleles, and one individual (0.5%) possessed five alleles (Figure 3a). Five of 14 island populations contained at least one individual with more than two MHC alleles. However, there was no obvious pattern in the proportion of individuals with different *A*_i_ values across islands (Figure 3b–k).

**Table 2:** Summary of microsatellite and MHC variability in *Podarcis erhardii* populations sampled from the 14 islands in the present study

Island code	*H*_e_ a	AR_msat_ b	*A*_i_ c	AR_MHC_ d	Θ_π_ e	Θ_k_ f	A^g^	P^h^	>2^i^
AK	0.58	2.72	1.00	2.05	11.18	1.96	4	1	0
AN	0.4	1.94	1.00	1.15	9.94	1.57	4	1	0
DA	0.61	2.76	1.58	3.12	13.33	8.17	13	2	5
KE	0.61	2.98	1.23	2.32	15.56	4.18	7	1	3
MP	0.39	1.81	1.24	1.43	7.67	1.86	5	2	3
GL	0.64	2.78	1.21	1.70	8.54	2.30	6	2	2
IR	0.77	3.51	1.23	2.67	10.53	4.18	7	1	3
KO	0.58	2.58	1.20	3.05	14.67	8.47	10	6	3
MA	0.58	2.45	1.00	0.31	2.25	2.20	2	3	0
SK	0.75	3.33	1.13	2.68	10.85	2.73	6	2	2
KP	0.51	2.26	1.15	1.57	18.29	2.20	5	1	1
MK	0.60	2.74	1.25	1.33	11.95	2.20	5	0	2
NX	0.77	3.59	1.35	2.53	15.16	3.92	8	2	3
OV	0.55	2.56	1.13	1.96	10.46	1.32	4	0	2

a Average expected microsatellite heterozygosity.

b Rarefacted estimates of microsatellite allelic richness averaged across loci.

c Average number of MHC alleles per individual (proxy for heterozygosity).

d Rarefacted estimates of MHC allelic richness averaged across loci.

e Estimates of theta based on the mean number of pairwise differences.

f Estimates of theta based on the number of segregating sites.

g Number of MHC alleles in each population.

h Number of private MHC alleles in each population.

i Number of individuals with more than two MHC alleles.

Both measures of MHC variability (*A*_i_ and AR_MHC_) were positively correlated with one another (*R* ^2^ = .36, *p* < .05; Table S1). AR_MHC_ was also positively correlated with AR_msat_ (*R* ^2^ = .34, *p* < .05) and *H*_e_ (*R* ^2^ = .29, *p* < .05). However, there was no significant association between *H*_e_ and *A*_i_. Both θ_π_ (2.25–18.29) and θ_K_ (1.32–8.47) ranged widely across island populations but showed no correlation with one another. In contrast, *A*_i_ and AR_MHC_ were positively correlated with θ_K_ (*R* ^2^ = .42, *p* < .05; *R* ^2^ = .54, *p* < .05), but only AR_MHC_ showed a positive correlation with θ_π_ (*R* ^2^ = .35, *p* < .05). There was no significant correlation between the number of individuals sequenced within each island (N_MHC_) and either AR_MHC_ or P, indicating that the sample sizes within islands were sufficient to give an unbiased estimate MHC diversity.

An examination of the distribution of MHC alleles by island provides evidence of substantial interisland differentiation (Figure 2). Of the 39 unique MHC alleles, 22 were restricted to single islands (private alleles). Only one allele (Poer‐DAB*267) was found in all four island groups and none were found on every island. The remaining alleles were generally spread across two or more fragmentation groups although one (Poer‐DAB*43) was restricted to islands within the Naxos group. The majority (84%) of pairwise genetic distances (*F*_ST_) between islands were also significant, reflecting substantial genetic MHC and microsatellite differentiation between islands. However, Mantel tests did not detect any evidence of a significant isolation by distance effect (*F*_1,89_ = 0.96, *df* = 2, *p* = .33).

Although an initial analysis of Tajima's *D* on the full dataset found no evidence of selection in the 14 islands surveyed, a sliding window approach identified six populations (Daskalio (DA), Keros (KE), Koufonnissi (KO), Naxos (NX), Ovriokastro (OV), and Schoinoussa (SK)) with a significantly positive Tajima's *D* statistic (*D* = 2.37–3.32, *p* < .05), consistent with a pattern of balancing selection and/or past bottleneck (Fig. S4). Amino acid residues exhibiting signatures of selection were all found within the PBR (Miller et al., 2005) (Fig. S5). Islands with signatures of balancing selection had significantly greater AR_MHC_ (*t* = 3.37, *df* = 12, *p* < .05) than islands that did not. In contrast, there were no significant differences in *A*_i_ (*t* = 1.76, *df* = 12, *p* > .05). Unfortunately, the limited number of microsatellite loci precluded any reliable estimate of Ne as the upper bound of the 95% confidence intervals for each estimate was nearly always infinity.

**Figure 4:**
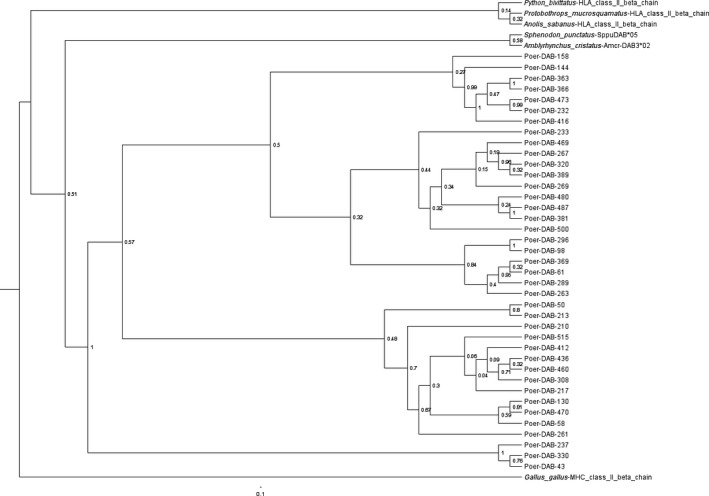
Phylogeny of major histocompatibility complex class II β (exon 2) in *Podarcis erhardii* and related taxa. The numbers at each node represent posterior probability support, and branch lengths are proportional to number of base pair substitutions

In general, we found little evidence for recombination between alleles. Only one significant recombination event was detected in the program MaxChi between sequences Poer‐DAB*50, Poer‐DAB*480, and Poer‐DAB*369 (*p* = .047). However, parental relationships between these alleles were unclear. Phylogenetic analysis provide strong support for the monophyly of *P. erhardii* MHC alleles relative to other published squamate reptile MHC class II β ‐1 subunit sequences (Glaberman, Du Pasquier, & Caccone, 2008; Miller, Belov, & Daugherty, 2006). Although the phylogeny revealed four distinct clusters of alleles, support for all but one of these clades was weak (<50%; Figure 4).

### 3.2 Comparison of mitochondrial, microsatellite, and MHC variability

AMOVA analyses revealed that much of the covariance in the mitochondrial data (79.89%) is structured by island fragmentation group (Table 3). In contrast, most of the covariance in microsatellite and MHC datasets is found within populations (islands) (70.78% and 68.78% respectively). However, both microsatellite and MHC marker sets exhibit a significant among‐population (island) differentiation component that exceeds the magnitude observed in the mitochondrial dataset. The among‐population component observed for the microsatellite data was comparable to that observed for the MHC (30.47% vs. 28.96%) and reflect the overall distribution of pairwise MHC and microsatellite F_ST_ values between islands (Fig. S6). BAYESASS analyses indicated little to no contemporary gene flow between all pairs of populations (islands).

**Table 3:** An analysis of molecular variance (AMOVA) of Aegean island populations of *P. erhardii* based on mitochondrial, microsatellite, and MHC variation

AMOVA				
	*df*	SS	Percentage covariance	*p*‐Value
Mitochondrial DNA				
Among groups	4	218.2	79.89	<.001
Among Pops.	12	25.2	8.26	<.001
Within Pops.	159	40.6	11.89	<.001
Total	175	284		
Microsatellite				
Among groups	3	71.74	−1.25	.63
Among Pops.	12	341.08	30.47	<.001
Within Pops.	732	984.9	70.78	<.001
Total	809	1515.96		
MHC				
Among groups	3	169.63	2.26	.24
Among Pops.	10	497.35	28.96	<.001
Within Pops.	235	1357.67	68.78	<.001
Total	248	2024.64		

### 3.3 Effects of island area and age on microsatellite and MHC variation

**Figure 5:**
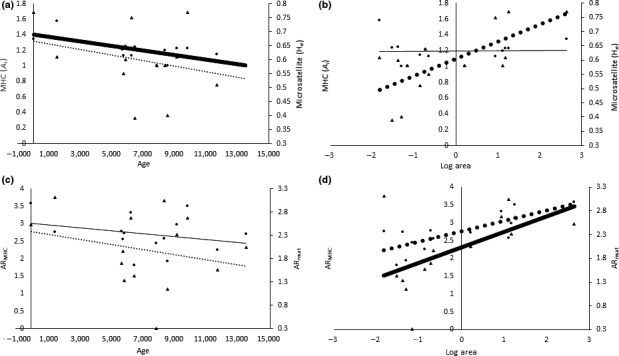
Regression of either island age or area against MHC and microsatellite variability. (a) Island age versus *A*_i_ (*p* = .0058, *R* ^2^ = .44) and *H*_e_ (*p* = .003, *R* ^2^ = .50). (b) The log of island area versus AR_msat_ and AR_MHC_ (*p* = .039, *R* ^2^ = .25), and AR_msat_ (*p* = .0016, *R* ^2^ = .54). (c) Island age versus *A*_i_ (*p* = .39, *R* ^2^ = −.015) and *H*_e_ (*p* = .31, *R* ^2^ = .01). (d) The log of island area versus AR_MHC_. (*p* = .903, *R* ^2^ = −.082) AR_msat_ (*p* = .62, *R* ^2^ = −.06). Regression lines for MHC are shown with a solid line and for microsatellite variation with a dotted line. Thickened lines indicate a significant relationship

**Figure 6:**
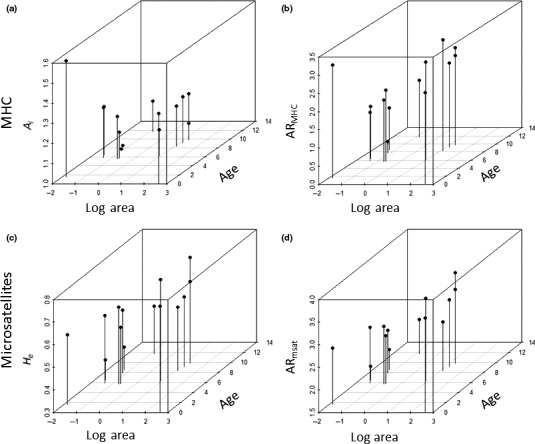
Scatter plots of *Podarcis erhardii* microsatellite and MHC variation in relation to island age and log Area. (a) *A*_i_ (*A*_i_ × Age *R* ^2^ = −.483; *A*_i_ × Log area *R* ^2^ = .001). (b) AR_MHC_ (AR_MHC_ × Age *R* ^2^ = −.063; AR_MHC_ × Log area = 0.308). (c) *H*_e_ (*H*_e_ × Age *R* ^2^ = −.143; *H*_e_ × Log area *R* ^2^ = .481). (d) AR_msat_ × Log area = 0.624; AR_msat_ × Age *R* ^2^ = −.2932)

Results from stepwise removal of variables from a fully saturated model agreed highly with models identified using a best subset regression analysis. Both bivariate and multiple regression analyses suggest that island area and age have shaped patterns of genetic variability. Microsatellite heterozygosity (*H*_e_; *p* < .05; AIC = −25.33) and allelic richness (AR_msat_; *p* < .01; AIC = 12.98) increased significantly with island area (Figures 5 and 6; Tables 4 and S2) but showed no relationship to age. In contrast, *A*_i_ was negatively associated with island age (*p* < .05; AIC = 18.51) but showed no relationship to island area. AR_MHC_ was also negatively associated with age (*p* < .05) and positively related to area (*p* < .05). This model also included a significant interaction between age and area (*p* < .05; AIC = 25.33). Genetic distance as measured by segregating sites (θ_k_) increased with island size (*p* < .05; AIC = 62.38) and the interaction of age and area. After applying the Holm multiple hypothesis correction, neither θ_π_ nor P showed any significant association with either island area or age or their interaction with one another. Two islands (NX, DA) were detected as potential outliers owing to their relatively high values of MHC variation. When Naxos and Daskalio were removed from the analysis, we found that the decline in *A*_i_ with island age, and the increase in θ_k_ were no longer significant. While AR_MHC_ was still positively associated with area, neither age or the area*age interaction term remained significant.

**Table 4:** Multiple regression analysis of the effects of island age and area on MHC and microsatellite variation in *Podarcis erhardii*. Models were explored by step‐wise removal of terms and selected by the lowest Akaike's Information Criterion (AIC) . Significant at * *p* < 0.05, ** *p* < 0.01

Model	Est	*SE*	p	AIC
MHC				
*A*_i_				
Age**	−3.50 × 10^−5^	8.70 × 10^−6^	2.00 × 10^−3^	
Log Area: Age	8.40 × 10^−6^	4.80 × 10^−6^	0.11	
Model				−18.51
AR_MHC_				
Age*	−1.02 × 10^−4^	4.13 × 10^−5^	3.30 × 10^−2^	
Log Area**	0.55	0.12	1.10 × 10^−3^	
Log Area: Age**	9.30 × 10^−5^	2.71 × 10^−5^	6.60 × 10^−2^	
Model				25.33
Θ_k_				
Age	−3.30 × 10^−4^	1.60 × 10^−4^	5.90 × 10^−2^	
Area*	1.06	0.45	4.20 × 10^−2^	
Log Area: Age*	2.70 × 10^−4^	1.00 × 10^−4^	2.60 × 10^−2^	
				62.38
Microsatellite				
*H*_e_				
Age	−1.20 × 10^−5^	6.80 × 10^−6^	0.10	
Log Area**	7.40 × 10^−2^	1.98 × 10^−2^	3.90 × 10^−3^	
Log Area: Age	6.00 × 10^−6^	4.40 × 10^−6^	0.20	
Model				−25.33
AR_msat_				
Age	−5.40 × 10^−5^	2.70 × 10^−5^	7.00 × 10^−2^	
Log Area**	0.36	7.80 × 10^−2^	3.90 × 10^−3^	
Log Area: Age	2.80 × 10^−5^	1.70 × 10^−5^	0.14	
Model				12.98

## 4 DISCUSSION

### 4.1 Patterns of MHC variability

The present study reveals significant population genetic structuring of MHC variability in *P. erhardii* island populations that is comparable to that of previous reports of other island reptile populations (Miller et al., 2010). Although most individuals that we sampled appeared to have only one or two alleles, we also detected eight individuals in four islands with three or more alleles, indicating the presence of copy number variation, as has been reported elsewhere (Eimes et al., 2011; Lighten, van Oosterhout, Paterson, et al., 2014).

Although high‐throughput sequencing can now capture sequence variation much more efficiently than traditional PCR amplification and cloning methods (Zagalska‐Neubauer, Babik, Stuglik, et al., 2010b), discriminating between alleles and artifacts remains a major challenge (Lighten, van Oosterhout, Paterson, et al., 2014; Lighten, van Oosterhout, & Bentzen, 2014). The interpretation of MHC data and other gene families is made especially complex by gene conversion events, PCR amplification bias, and sequence artifacts created through template switching during the PCR reaction (Bradley & Hillis, 1997; Cummings, McMullan, Joyce, & van Oosterhout, 2010; Paabo, Irwin, & Wilson, 1990; Saiki, 1989; Sommer, Courtiol, & Mazzoni, 2013). To tackle these challenges, the present study capitalized on a relatively new method for quantifying variation in complex multigene families like the MHC (Lighten, van Oosterhout, Paterson, et al., 2014) and circumvents some of the problems posed by earlier allele validation threshold methods (Babik et al., 2009; Nadachowska‐Brzyska, Zieliński, Radwan, & Babik, 2012; Zagalska‐Neubauer, Babik, Stuglik, et al., 2010b), leading to greater confidence in the number of alleles detected.

### 4.2 Relative effects of selection, drift, migration, and recombination on MHC variation

It is generally accepted that parasite‐mediated selection is responsible for maintaining MHC variability in host populations (Spurgin & Richardson, 2010) and that this variability may be maintained even in the face of severe population bottlenecks (Aguilar et al., 2004; van Oosterhout, Weetman, & Hutchinson, 2006; van Oosterhout, 2009; Oliver & Piertney, 2012; Newhouse & Balakrishnan, 2015; but see Miller et al., 2010; Eimes et al., 2011; Hoglund, Wengstrom, Rogeli, & Meyer‐Lucht, 2015; Zhang et al., 2016). However, other studies have shown that MHC variation can decline dramatically in small populations (e.g., Eimes et al., 2011; Hoglund et al., 2015; Miller et al., 2010; Zhang et al., 2016), underlining the importance of drift in some systems. Ultimately, the relative impact of selection and drift on MHC diversity will depend not only on the mode of parasite‐mediated selection, but also on the magnitude of gene flow and recombination.

In this study, we capitalized on the Cyclades island system to examine the relative impact of all four evolutionary forces in a suite of island populations of *P. erhardii* subject to population bottlenecks of differing magnitude and duration. Our first hypothesis posited that variability in the MHC in island populations of *P. erhardii* was maintained through parasite‐mediated selection and that this variation would persist even in populations subject to severe population bottlenecks. Alternatively, we hypothesized that drift was the predominant force and that neither selection, migration nor recombination was sufficient to overcome its cumulative effects. In keeping with this second hypothesis, we find that patterns of MHC variation appear to be largely governed by drift. Furthermore, the lack of a significant effect of isolation by distance, negligible migration and high proportion of private alleles suggest that gene flow between islands is negligible, as has been suggested previously (Foufopoulos & Ives, 1999). Furthermore, at least over the short MHC sequences examined in the present study, recombination appeared to be largely absent.

Significantly, positive values of Tajima's *D* were observed on six of the 14 islands examined in the present study, consistent with patterns expected under balancing selection. Although population bottlenecks can also yield a positive Tajima's *D* (Tajima, 1989), those islands with significantly positive values were not those necessarily subject to the most severe bottlenecks. However, MHC F_ST_ values were not lower than those of microsatellites, as might be expected under a scenario of broad scale balancing selection (Rico, Morris‐Pocock, Zigouris, Nocera, & Kyle, 2015), suggesting that the effects of balancing selection in this system are weak. Equally, our data do not support a scenario of spatially heterogeneous parasite‐mediated selection as the among‐population component of MHC variability does exceed that of microsatellite data (*cf*. Loiseau et al., 2009). Instead, findings from our study support a scenario where drift is the prevailing force (cf. Runemark et al., 2010; Runemark et al. 2012) and where selection, immigration, and recombination make relatively little contribution to overall patterns of contemporary MHC variability.

### 4.3 Effects of island area and age

Consistent with the effects of drift, regression analyses presented here also demonstrate that microsatellite heterozygosity (*H*_e_), microsatellite allelic richness (AR_msat_,), and MHC allelic richness (AR_MHC_) are all positively related to island area, supporting the hypothesis that smaller islands have lost both neutral and MHC variation through drift. MHC variability (*A*_i_ and AR_MHC_) was also found to decline significantly with island age. Although this decline could be simply due to the cumulative effects of drift, we cannot rule out the possibility that lizards on older islands have lower copy numbers. The decline in MHC variation could also be due to relaxed balancing selection in which parasite‐mediated selection declines over time (Lahti et al., 2009). Under this model, we would expect to find signatures of balancing selection in younger islands but not in older islands. Our data, however, do not appear to support this hypothesis as island populations with significant signatures of selection span a range of different ages.

Although previous studies reported a negative relationship between AR_msat_ and island age (Hurston et al., 2009), this effect disappeared when we corrected for sample size. Such an apparent lack of effect of island age on microsatellite variability is consistent with the high mutation rate at these loci (μ ≈ 3 × 10^−3^ to 1 × 10^−4^/locus/generation; for example, Hohoff et al., 2006; Tesson et al., 2013), which is four to five orders of magnitude higher than that of genomic single nucleotide polymorphic mutations. For each microsatellite locus, hundreds or perhaps even thousands of new alleles will have been generated (and lost) in each population during the 20,000 years as the sea level rise that formed this population complex (Foufopoulos & Ives, 1999). The genetic diversity of these microsatellite loci is therefore in a mutation‐drift balance that long‐since reached its equilibrium (Fig. S7), and which is determined by the effective population size (and hence area of the island), as well as the mutation rate of the locus. Consequently, island age no longer affects diversity at these loci. In contrast, the much slower evolving MHC loci may not have yet reached their mutation‐selection‐drift equilibrium, which could explain why we found a significant decline in MHC variation with island age.

Lastly, removal of two potential outliers (NX, DA) eliminated the effect of age on MHC variability, indicating that the effective age on the MHC is highly dependent on these two islands. Their relatively high levels of MHC variation could be due to a number of reasons. Firstly, NX is an island that constitutes the largest remaining fragment of the ancestral land mass that gave rise to the present island system. As such, this island has never undergone a historical bottleneck and therefore may have simply retained greater levels of ancestral variation than other islands in the system. Conversely, DA is a very young island that is separated by a short distance from a much larger land mass (Antikeros) that could have served as a potential source of migrants. Either way, one or both factors could potentially limit the effects of drift and explain why this island displayed unexpectedly high levels of MHC variation.

## 5 CONCLUSIONS

In this study, we capitalize on a suite of islands of known bottleneck history to unravel the effects of drift, selection, migration, and recombination on MHC variation in island populations of Aegean wall lizard *P. erhardii*. We show that 454 sequencing, and use of a novel dynamic threshold approach for assigning alleles, can be used to reliably quantify MHC variability. Our findings show that MHC variation is determined largely by genetic drift despite signatures of balancing selection in some islands. Furthermore, immigration and recombination appear to have contributed very little to overall patterns of MHC diversity. Studies of MHC variability in squamate reptiles have been limited to a handful of taxa so that the present study adds substantially to our current knowledge of reptilian MHC. Future work should seek to tie assays of MHC variation with parasite loads and physiological assays of immune function in island populations subject to different bottleneck histories. Together this information will provide greater insight into the relationship between pathogen pressure and variation at the MHC and provide a means for directly assessing the functional consequences of losses in adaptive variation in immunity loci on disease resistance.

## CONFLICT OF INTEREST

None declared.

## DATA ACCESSIBILITY

Raw 454 sequence reads have been submitted to the BioProject portion of National Center for Biotechnology Information (NCBI), submission number—SUB2464415, project ID—PRJNA378300. The trimmed sequencing data, read depths by sample, and list of alleles found in each individual have been submitted to Dryad in a single data package.
