# Supplementary material for: The effects of historical fragmentation on major histocompatibility complex class II β and microsatellite variation in the Aegean island reptile, Podarcis erhardii
Source: Ecol Evol. 2017 May 18;7(13):4568–81. doi: 10.1002/ece3.3022 (PMC5496512; doi:10.1002/ece3.3022)
Supplement: Supplementary file 1 [file ECE3-7-4568-s001.docx]

**Supplementary figure S1.** Nucleotide alignment of all 39 *Podarcis erhardii* MHC alleles typed in the present study.

43 GAGTGCCACTTCTTCAACGGGACGCAGCGGATCCGCCTCCTAATTAGGTACTTCTACGACCGGCAGGAGGTCGACTACTTCGACAGCGACCGAGGGAAGTTCGTGGC

50 G......A.....C.......A........A...G.TT....AA...AT.C....TC.G..........C..CTC..............C.........G.......

58 G......A.....C.......A........G...G.TT....AA...AT.C....TC.A..........C..TCC..............C.........T.......

61 A......A.....C.......A........G...G.CT....CA...TT.C....TT.A..........C..CTC..............C.........T.......

98 G......A.....C.......A........G...G.TT....CA...TT.C....TT.A..........C..CTC..............C.........T.......

130 G......A.....C.......A........G...G.CT....CA...TT.C....TC.A..........C..GCC..............C.........T.......

144 G......A.....C.......A........G...G.CT....CA...TT.C....TT.A..........C..CTC..............C.........G.......

158 G......A.....C.......A........G...G.CT....CA...TT.C....TT.A..........C..CTC..............C.........T.......

210 G......A.....C.......A........A...G.TT....AA...AT.C....TC.A..........C..TCC..............C.........T.......

213 G......A.....C.......A........A...G.TT....AA...AT.C....TC.A..........C..TCC..............C.........T.......

217 G......A.....C.......A........A...G.TT....AA...AT.C....TC.A..........C..TCC..............C.........T.......

232 G......A.....C.......A........G...G.TA....GT...GC.G....TC.G..........C..TTC..............C.........G.......

233 G......A.....C.......A........G...G.CT....CA...TT.C....TT.A..........C..CTC..............C.........T.......

237 G......A.....T.......A........A...G.CT....AT...GT.C....AC.A..........G..GAC..............C.........T.......

261 G......A.....C.......A........A...G.TT....AA...AT.C....TC.A..........C..TCC..............C.........T.......

263 G......A.....C.......A........G...G.CT....CA...TT.C....TT.A..........C..CTC..............C.........T.......

267 G......A.....C.......A........G...G.CT....CA...TT.C....TT.A..........C..GCC..............C.........T.......

269 G......A.....C.......A........G...G.CT....CA...TT.C....TC.G..........C..GCC..............C.........T.......

289 G......A.....C.......A........G...G.CT....CA...TT.C....TT.A..........C..CTC..............C.........T.......

296 G......A.....C.......A........G...G.CT....CA...TT.C....TT.A..........C..CTC..............C.........T.......

308 G......A.....C.......A........A...G.TT....AA...AT.C....TC.A..........C..CTC..............C.........T.......

320 G......A.....C.......A........G...G.CT....CA...TT.T....TT.A..........C..GCC..............C.........T.......

330 G......A.....T.......A........A...G.CT....AT...GT.C....AC.A..........G..GAC..............C.........T.......

363 G......A.....C.......A........A...G.TA....GA...GC.C....TC.G..........C..TCC..............C.........T.......

366 G......A.....C.......A........A...G.TA....GA...GC.C....TC.G..........C..TCC..............C.........T.......

369 G......A.....C.......A........G...G.CT....CA...TT.C....TT.A..........C..CTC..............G.........T.......

381 G......A.....C.......A........G...G.CT....CA...TT.C....TC.A..........C..GCC..............C.........T.......

389 G......A.....C.......G........G...G.CT....CA...TT.C....TT.A..........C..GCC..............C.........T.......

412 G......A.....C.......A........A...G.TT....AA...AT.C....TC.A..........C..GCC..............C.........T.......

416 G......A.....C.......A........G...A.TA....GA...AT.C....TC.G..........C..TAT..............C.........G.......

436 G......A.....C.......A........A...G.TT....AA...AT.C....TC.A..........C..TCC..............C.........T.......

460 G......A.....C.......A........A...G.TT....AA...AT.C....TC.A..........C..TCC..............C.........T.......

469 G......A.....C.......A........G...G.CT....CA...TT.C....TT.A..........C..GCC..............C.........T.......

470 G......A.....C.......A........G...G.CT....CA...TT.C....TC.A..........C..TCC..............C.........T.......

473 G......A.....C.......A........G...G.TA....GT...GC.G....TC.G..........C..TTC..............C.........G.......

480 G......A.....C.......A........G...G.CT....CA...TT.C....TT.A..........C..GCC..............C.........T.......

487 G......A.....C.......A........G...G.CT....CA...TT.C....TC.A..........C..GCC..............C.........T.......

500 G......A.....C.......A........G...G.CT....CA...TT.C....TT.A..........C..CTC..............C.........T.......

515 G......G.....C.......A........A...G.TT....AA...AT.C....TC.A..........C..TCC..............C.........T.......

43 GGTGGCTGAAATAATGGAGTCCGACGTCAAGAGCTGGAACCAGGACAAGGAGCGCCTGCAGACCGCGATGGCCGATGTGGATCGCTTCTGCCGCCACAACTATGGGATCTACGAG

50 T.....T...C.GGG....C..GAC.T.G..AGA.................CAT.....GGAGGAAG.A.G..GCC......GT..T.T.C.GGC..A..T.CGAGAT.CACGCG

58 T.....T...C.GGG....C..GAC.C.A..GCA.................CAT.....GGAGAAAC.T.G..GAC......CG..T.T.C.GGC..A..T.CGAGAT.GACGCG

61 T.....T...C.GGG....C..GAC.C.A..GCA.................CAT.....GCAACTGG.T.G..GAC......GT..T.T.C.GGC..A..T.CGGGAT.CACGCG

98 T.....T...C.GGG....C..ATA.T.A..GCA.................CAT.....GCAACTGG.T.G..GAC......TC..T.T.C.GGA..A..T.CGGGGT.CTCGAT

130 T.....T...C.GGG....C..GAC.C.A..GCA.................CAT.....GGAGAAAC.T.G..GAC......CG..T.T.C.GGC..A..T.CGAGAT.GACGCG

144 T.....T...C.GGG....C..GAC.T.A..AGA.................CCT.....GGAGCGCG.T.G..GAC......GT..T.T.C.GGC..A..T.CGGGGT.CTCGAT

158 T.....T...C.GGG....C..GCA.T.A..AGA.................CGC.....GGAGAAAC.T.G..GAC......GT..T.T.C.GGC..A..T.CGGGAT.CACGCG

210 T.....T...C.GGG....C..GAC.T.G..AGA.................CAT.....GGAGAAAC.T.G..GAC......CG..T.T.C.GGA..A..T.CGAGAT.GACGCG

213 T.....T...C.GGG....C..GAC.T.G..AGA.................CAT.....GGAGGAAG.A.G..GCC......GT..T.T.C.GGC..A..T.CGAGAT.CACGCG

217 T.....T...C.GGG....C..GAC.C.A..GCA.................CAT.....GGAGAAAC.T.G..GAC......CG..T.T.C.GGC..A..T.CGAGAT.GACGCG

232 T.....T...C.GGG....C..GAC.T.A..AGA.................CCT.....GGAGCGCG.T.G..GAC......GT..T.T.C.GGC..A..T.CGGGGT.CTCGAT

233 T.....T...C.GGG....C..ATA.T.A..GCA.................CAT.....GGAGGAAG.A.G..GAC......TC..T.T.C.GGC..A..T.CGGGAT.CACGCG

237 G.....T...A.AAT....T..GAC.T.A..AGA.................GCG.....AGACCGCG.T.G..GAT......CG..T.T.C.GCC..A..T.TGGGAT.TACGAG

261 T.....T...C.GGG....C..GAC.C.A..GCA.................CAT.....GGAGAAAC.T.G..GAC......CG..T.T.C.GGA..A..T.CGAGAT.GACGCG

263 T.....T...C.GGG....C..ATA.T.A..GCA.................CAT.....GCAACTGG.T.G..GAC......GT..T.T.C.GGC..A..T.CGGGAT.CACGCG

267 G.....T...C.GGG....C..ATA.T.A..GCA.................CAT.....GGAGGAAG.A.G..GAG......TC..T.T.C.GGC..A..T.CGGGAT.CACGCG

269 T.....T...C.GGG....C..ATA.T.A..GCA.................CAT.....GGAGGAAG.A.G..GAG......TC..T.T.C.GGC..A..T.CGGGAT.CACGCG

289 T.....T...C.GGG....C..GAC.C.A..GCA.................CAT.....GCAACTGG.T.G..GAC......GT..T.T.C.GGC..A..T.CGGGAT.CACGCG

296 T.....T...C.GGG....C..ATA.T.A..GCA.................CAT.....GCAACTGG.T.G..GAC......TC..T.T.C.GGA..A..T.CGGGGT.CTCGAT

308 T.....T...C.GGG....C..GAC.C.A..GCA.................CAT.....GGAGAAAC.T.G..GAC......CG..T.T.C.GGC..A..T.CGAGAT.CACGCG

320 G.....T...C.GGG....C..ATA.T.A..GCA.................CAT.....GGAGGAAG.A.G..GAG......TC..T.T.C.GGC..A..T.CGGGAT.CACGCG

330 G.....T...A.AAT....T..GGC.T.A..AGC.................GCG.....AGACCGCG.T.G..GAT......CG..T.T.C.GCC..A..T.TGGGAT.TACGAG

363 G.....T...C.GGG....C..GAC.C.A..AGA.................CGT.....GGGGTGTG.T.T..AAC......TC..T.T.C.GGA..A..T.CGGGGT.CTCGAT

366 G.....T...C.GGG....C..GAC.C.A..AGA.................CGT.....GGGGTGTG.T.T..AAC......CG..T.T.C.GGA..A..T.CGGGGT.CTCGAT

369 T.....T...C.GGG....C..GAC.C.A..GCA.................CAT.....GCAACTGG.T.G..GAC......GT..T.T.C.GGC..A..T.CGGGAT.CACGCG

381 T.....T...C.GGG....C..ATA.T.A..AGA.................CCT.....GGAGCGAG.A.G..GCG......TC..T.T.C.GGC..A..T.CGGGGG.ATCGAG

389 G.....T...C.GGG....C..ATA.T.A..GCA.................CAT.....GGAGGAAG.A.G..GAG......TC..T.T.C.GGC..A..T.CGGGAT.CACGCG

412 T.....T...C.GGG....C..GAC.C.A..GCA.................CAT.....GGAGAAAC.T.G..GAC......CG..T.T.C.GGC..A..T.CGAGAT.GACGCG

416 T.....T...C.GGC....C..GAC.T.A..AGA.................CAT.....GGGGCGAG.T.G..GCC......GT..T.T.C.GGC..A..T.CGGGGG.TTCGAG

436 T.....T...C.GGG....C..GAC.C.A..GCA.................CAT.....GGAGAAAC.T.G..GAC......CG..T.T.C.GGC..A..T.CGAGAT.CACGCG

460 T.....T...C.GGG....C..GAC.C.A..GCA.................CAT.....GGAGAAAC.T.G..AAC......AT..G.C.G.ACA..T..G.GATCCA.GCG---

469 T.....T...C.GGG....C..ATA.T.A..GCA.................CAT.....GGAGGAAG.A.G..GAG......TC..T.T.C.GGC..A..T.CGGGAT.CACGCG

470 T.....T...C.GGG....C..GAC.C.A..GCA.................CAT.....GGAGAAAC.T.G..GAC......CG..T.T.C.GGC..A..T.CGAGAT.GACGCG

473 T.....G...C.GGG....C..GAC.T.A..AGA.................CCT.....GGAGCGCG.T.G..GAC......GT..T.T.C.GGC..A..T.CGGGGT.CTCGAT

480 T.....T...C.GGG....C..GAC.T.G..AGA.................CAT.....GGAGGAAG.A.G..GAG......TC..T.T.C.GGC..A..T.CGGGAT.CACGCG

487 T.....T...C.GGG....C..ATA.T.A..AGA.................CAT.....GGAGCGAG.A.G..GCG......TC..T.T.C.GGC..A..T.CGGGGG.ATCGAG

500 T.....T...C.GGG....C..ATA.T.A..GCA.................CAT.....GGAGGAAG.A.G..GAG......TC..T.T.C.GGC..A..T.CGGGAT.CACGCG

515 T.....T...C.GGG....C..GAC.C.A..GCA.................CAT.....GGAGAAAC.T.G..GAC......CG..T.T.C.GGC..A..T.CGAGAT.GACGCG

| **Supplementary figure S2.** Amino acid alignment of MHC alleles identified by 454 sequencing of Aegean island populations of *Podarcis erhardii.* |
| --- |
| 43 ECHFFNGTQRIRLLIRYFYDRQEVDYFDSDRGKFVAVAEIMESDVKSWNQDKERLQTAMADVDRFCRHNYGIYE  50 E.H.S..T..IRF.NRY.FG...LL....D...V.....LG.PDVER.....DI.RRKKAA..VFCRHNYEIHA  58 E.H.S..T..VRF.NRY.FD...LS....D...F.....LG.PDAKA.....DI.RRNMAD..RFCRHNYEIDA  61 K.H.S..T..VRL.HSY.FD...LL....D...F.....LG.PDAKA.....DI.RNWMAD..VFCRHNYGIHA  98 E.H.S..T..VRF.HSY.FD...LL....D...F.....LG.PIVKA.....DI.RNWMAD..SFCRNNYGVLD  130 E.H.S..T..VRL.HSY.FD...LA....D...F.....LG.PDAKA.....DI.RRNMAD..RFCRHNYEIDA  144 E.H.S..T..VRL.HSY.FD...LL....D...V.....LG.PDVKR.....DL.RSAMAD..VFCRHNYGVLD  158 E.H.S..T..VRL.HSY.FD...LL....D...F.....LG.PAVKR.....DA.RRNMAD..VFCRHNYGIHA  210 E.H.S..T..IRF.NRY.FD...LS....D...F.....LG.PDVER.....DI.RRNMAD..RFCRNNYEIDA  213 E.H.S..T..IRF.NRY.FD...LS....D...F.....LG.PDVER.....DI.RRKKAA..VFCRHNYEIHA  217 E.H.S..T..IRF.NRY.FD...LS....D...F.....LG.PDAKA.....DI.RRNMAD..RFCRHNYEIDA  232 E.H.S..T..VRY.VRQ.FG...LF....D...V.....LG.PDVKR.....DL.RSAMAD..VFCRHNYGVLD*  233 E.H.S..T..VRL.HSY.FD...LL....D...F.....LG.PIVKA.....DI.RRKKAD..SFCRHNYGIHA  237 E.H.F..T..IRL.IRY.YD...VD....D...F.....IM.SDVKR.....ER.QTAMAD..RFCRHNYGIYE  261 E.H.S..T..IRF.NRY.FD...LS....D...F.....LG.PDAKA.....DI.RRNMAD..RFCRNNYEIDA  263 E.H.S..T..VRL.HSY.FD...LL....D...F.....LG.PIVKA.....DI.RNWMAD..VFCRHNYGIHA  267 E.H.S..T..VRL.HSY.FD...LA....D...F.....LG.PIVKA.....DI.RRKKAE..SFCRHNYGIHA**  269 E.H.S..T..VRL.HSY.FG...LA....D...F.....LG.PIVKA.....DI.RRKKAE..SFCRHNYGIHA  289 E.H.S..T..VRL.HSY.FD...LL....D...F.....LG.PDAKA.....DI.RNWMAD..VFCRHNYGIHA  296 E.H.S..T..VRL.HSY.FD...LL....D...F.....LG.PIVKA.....DI.RNWMAD..SFCRNNYGVLD  308 E.H.S..T..IRF.NRY.FD...LL....D...F.....LG.PDAKA.....DI.RRNMAD..RFCRHNYEIHA  320 E.H.S..T..VRL.HSY.FD...LA....D...F.....LG.PIVKA.....DI.RRKKAE..SFCRHNYGIHA**  330 E.H.F..T..IRL.IRY.YD...VD....D...F.....IM.SGVKS.....ER.QTAMAD..RFCRHNYGIYE  363 E.H.S..T..IRY.DRH.FG...LS....D...F.....LG.PDAKR.....DV.RGVMSN..SFCRNNYGVLD  366 E.H.S..T..IRY.DRH.FG...LS....D...F.....LG.PDAKR.....DV.RGVMSN..RFCRNNYGVLD  369 E.H.S..T..VRL.HSY.FD...LL....E...F.....LG.PDAKA.....DI.RNWMAD..VFCRHNYGIHA  381 E.H.S..T..VRL.HSY.FD...LA....D...F.....LG.PIVKR.....DL.RSEKAA..SFCRHNYGGIE  389 E.H.S..A..VRL.HSY.FD...LA....D...F.....LG.PIVKA.....DI.RRKKAE..SFCRHNYGIHA  412 E.H.S..T..IRF.NRY.FD...LA....D...F.....LG.PDAKA.....DI.RRNMAD..RFCRHNYEIDA  416 E.H.S..T..VHY.DRY.FG...LY....D...V.....LA.PDVKR.....DI.RGEMAA..VFCRHNYGGFE  436 E.H.S..T..IRF.NRY.FD...LS....D...F.....LG.PDAKA.....DI.RRNMAD..RFCRHNYEIHA  460 E.H.S..T..IRF.NRY.FD...LS....D...F.....LG.PDAKA.....DI.RRNMAN..ICRHNYEIHA-  469 E.H.S..T..VRL.HSY.FD...LA....D...F.....LG.PIVKA.....DI.RRKKAE..SFCRHNYGIHA**  470 E.H.S..T..VRL.HSY.FD...LS....D...F.....LG.PDAKA.....DI.RRNMAD..RFCRHNYEIDA  473 E.H.S..T..VRY.VRQ.FG...LF....D...V.....LG.PDVKR.....DL.RSAMAD..VFCRHNYGVLD*  480 E.H.S..T..VRL.HSY.FD...LA....D...F.....LG.PDVER.....DI.RRKKAE..SFCRHNYGIHA  487 E.H.S..T..VRL.HSY.FD...LA....D...F.....LG.PIVKR.....DI.RSEKAA..SFCRHNYGGIE  500 E.H.S..T..VRL.HSY.FD...LL....D...F.....LG.PIVKA.....DI.RRKKAE..SFCRHNYGIHA  515 E.R.S..T..IRF.NRY.FD...LS....D...F.....LG.PDAKA.....DI.RRNMAD..RFCRHNYEIDA |

* Asterisks denote identical amino acid sequences.

**Supplementary figure S3.** Average number of MHC alleles per individual (*A_i_*) plotted against sequencing read depth (R^2^ = 0.001).

**Supplemental figure S4.** Plot of Tajima’s D statistic in relation to nucleotide position within the putative binding site of the *Podarcis erhardii* MHC using a sliding window of three base pairs. The locations in the alignment where significant positive Tajima’s D statistics were observed are indicated with a bar above the graph.


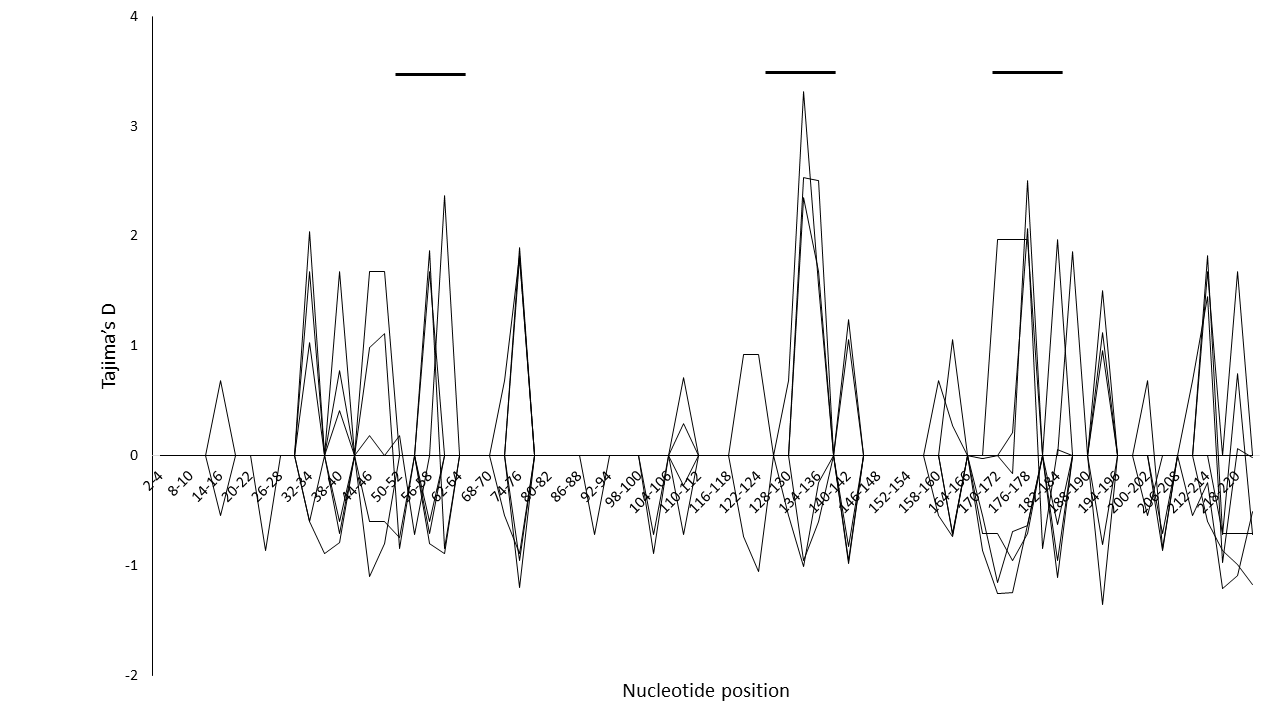


**Supplementary figure S5.** MHC sequences derived from the cDNA from a single *Podarcis erhardii* sample. The top block encompasses the β – 1 region within exon 2 and contains the putative peptide binding region. The underlined amino acids correspond to the nucleotide locations of the primers used for 454 sequencing. The lower block is part of exon 3 containing the β – 2 region.

*Podarcis erhardii* ????????LFQSKGECHFSNGTQ-------RIRFLNRYFFDRQELSYFDSDRGKFVAVAELGEPDVERWNQDKDILRRNMADVDRFCRNNYEIDAPFAQSRR

*Sphenodon spp.* ????????.L.W.FQ.LYT..MG-------.....Y.CYWG...FVR......L.EP.T...R..A.G..RQPET.QYYR.ALE...PH..GVIES.TVQ.C

*Amblyrhynchus cristatus* GAEPPEHF.I...Y..SLTAAAAGSRPEDVEV..VH.FIY..E..AR...A..EYE...A.....ARY..GQ.EV.D.RR.E..A...H..GVYE...RK.L

*Podarcis erhardii* VQPKVKITPTDNVESSPHNTLLICTVNRFFPAGIEIKWLRNGEEE-PKAWTTDLIRNGDWTFHIEVMLETKPERGDVYT

*Sphenodon spp.* .E.R...S..Q-LD.LH.P...V.S.TG.Y..E.....F...Q..TAG.VS.E.LQ......Q.L.....A.R......

*Amblyrhynchus cristatus* ....L..SA.E-HD..SQ......N.A..W..E.....F...K..-EDVM..S.........Q.Q........K.....

**Supplementary figure S6.** Scatterplot of pairwise F_ST_ values derived from MHC and microsatellite data (R^2^ = 0.067).


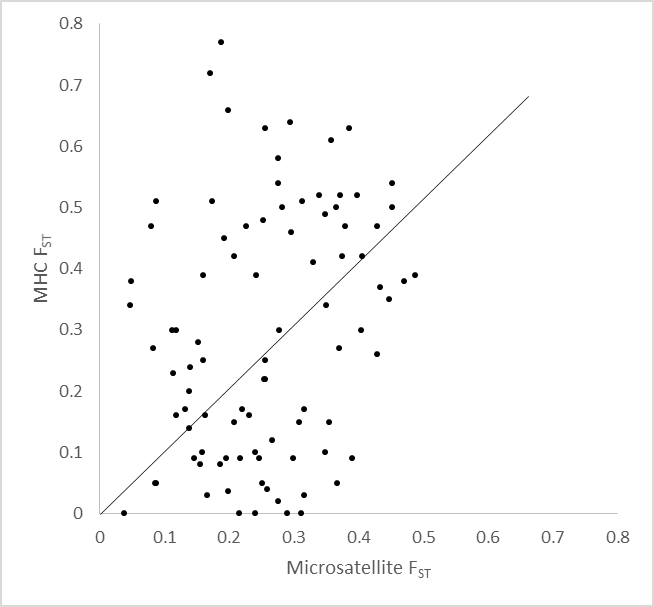


**Supplementary figure S7**. Mutation-drift equilibrium of a microsatellite locus with *µ* = 5x10^-4^ at a locus with a maximum of 50 alleles that is mutating according to a two-phase mutation model. Equilibrium is reached within 20,000 generations for populations with effective size

*N_e_* of 25,000 and at 5,000 generations for populations with *N_e_* values of 2,500 and 250.

| **Supplementary table S1.** Bivariate regressions between island and genetic variables; significant t-values (P < 0.05) are reported in bold face. | | | | | | | | | | |
| --- | --- | --- | --- | --- | --- | --- | --- | --- | --- | --- |
|  | Age | Log Area | Distance | H_e_ | AR_msat_ | *A_i_* | AR_MHC_ | N_MHC_ | Private | Θ_k_ |
| Age |  |  |  |  |  |  |  |  |  |  |
| Log Area | -0.15 |  |  |  |  |  |  |  |  |  |
| Distance | **2.94** | 2.04 |  |  |  |  |  |  |  |  |
| H_e_ | -1.09 | **3.39** | 0.98 |  |  |  |  |  |  |  |
| AR_msat_ | -1.07 | **4.06** | 1.23 | **17.30** |  |  |  |  |  |  |
| *A_i_* | **-3.35** | 0.12 | **-3.35** | 1.12 | 1.20 |  |  |  |  |  |
| AR_MHC_ | -0.90 | **2.31** | -0.90 | **2.20** | **2.51** | **2.58** |  |  |  |  |
| N_MHC_ | **-4.02** | -0.44 | **-4.02** | 0.49 | 0.19 | **2.26** | 0.58 |  |  |  |
| P | -0.30 | 0.85 | 0.02 | -0.22 | -0.13 | 0.24 | 0.96 | 1.41 |  |  |
| Θ_k_ | -1.06 | 0.91 | -1.09 | 1.02 | 0.99 | **2.95** | **3.72** | 1.13 | **2.90** |  |
| Θ_π_ | 0.98 | 1.48 | 0.03 | 0.57 | 0.90 | 1.53 | **2.55** | 0.87 | -0.16 | 1.49 |
