# Supplementary material for: The effects of historical fragmentation on major histocompatibility complex class II β and microsatellite variation in the Aegean island reptile, Podarcis erhardii
Source: Ecol Evol. 2017 May 18;7(13):4568–81. doi: 10.1002/ece3.3022 (PMC5496512; doi:10.1002/ece3.3022)
Supplement: Supplementary file 2 [file ECE3-7-4568-s002.docx]

**Supplementary Methods**

Amplification of the MHC class II β– 1 subunit from *P. erhardii* cDNA

Total RNA was extracted from tissue obtained from the tail tip of an individual *P. erhardii* collected from Naxos (Cyclades, Greece) using the RNEasy kit (Qiagen, CA). First and second strand cDNA synthesis was carried out using the MINT cDNA kit (Evrogen, Russia). PCR amplification was performed using degenerate primers MHC2Ex2F2 and MHC2Ex3R2 (Miller *et al.* 2005) in 25 µL reactions under the following conditions: 15 - 30 ng cDNA, 1.5 mM MgCl_2_, 200 µM each dNTP and 0.4 µM each primer, for 30 cycles of 94°C for 30 s, 50°C for 20 s and 72°C for one minute. Target amplicons were cloned into a pCR2.1 vector using the TA Cloning Kit (Invitrogen, NY), sequenced using the BigDye Terminator Cycle Sequencing kit (version 3.1) and analyzed on an ABI 3100 automated sequencer.

MHC 454 sequencing and analysis

Individual MID barcodes of 10 bp in length were added to the forward species-specific primer (PodEx2F1) and combined with a universal reverse PodEx2R1 primer for initial amplification of 225 base pair fragment of exon 2. PCR amplification was performed in 50 µl reactions using 50 - 100 ng genomic DNA, 1X buffer, 1.5 mM MgCl_2_, 200 µM each dNTP, 0.2 µM each primer and 1.25 U of Taq polymerase (LifeTechnologies, Grand Island, NY). Cycle parameters comprised an initial denaturation at 95°C for five minutes, followed by 35 cycles of 94°C for 15 s, 53°C for one minute and 72°C for 30 s with a final step of 72°C for ten minutes. PCR products were purified using the Agencourt AMPure XP magnetic beads (Beckman-Coulter, Brea, CA), quantified using a NanoDrop spectrophotometer (Thomson Scientific, Wilmington, DE) and adjusted to approximately equimolar proportions prior to sequencing. In order to minimize the number of barcodes needed for 300 individuals, we generated two pools of 150 primer sets and incorporated an additional 10 bp MID barcode for each pool during library preparation. These two tagged libraries were combined and sequenced on a GS FLX 454 sequencer (Roche 454 Life Sciences, Basel, Switzerland). A bioinformatics pipeline was developed using a custom script in Python 2.7 (Python Software Foundation) to first segregate sequences from the two libraries and parse them into individual sub-libraries based on the incorporated MID sequences.

Justification for the DOC method

One difficulty in quantifying MHC variability using 454 data is separating true sequences from artifacts generated through sequencing error, base mis-incorporation, PCR chimera formation and cross-sample contamination (Zagalska-Neubauer *et al.* 2010a; Lighten *et al.* 2014a; b). Although previous studies have used a threshold approach to separating true alleles from artifacts (see Babik *et al.* 2009; Galan *et al.* 2010; Zagalska-Neubauer *et al.* 2010b; Nadachowska-Brzyska *et al.* 2012; Sommer *et al.* 2013), such approaches can bias estimates of MHC variation as they fail to take into account variability in read depth between individual amplicons (Lighten *et al.* 2014b). In order to quantify MHC variability, we therefore employed the Degree of Change (DOC) method developed by Lighten *et al.* (2014a; b) which makes use of the rate of change in cumulative read depth within each amplicon to estimate the number of alleles per individual (*A_i_*).

Comparison of mitochondrial, microsatellite and MHC variability

Mitochondrial and microsatellite data analyzed in the present study were previously published in Hurston et al. (2009). Although initially 27 microsatellite primer pairs from a range of lacertid lizards were tested on *P. erhardii*, only 13 primer sets amplified PCR products of the expected size (Hurston et al., 2009). Only six of these loci were found to be variable, one of which failed to contain a microsatellite motif, leaving us with five microsatellite loci. Overall, there were fifteen instances of significant deviations from Hardy-Weinberg equilibrium, the vast majority of which were found in a single locus T434. Of the islands included in this study, only one (Andreas) exhibited significant signatures of linkage disequilibrium at more than one pair of loci. Statistical analyses conducted with and without the T434 locus did not affect findings reported in Hurston et al. (2009) so we chose to retain this locus in the present study.

MHC phylogeny estimation

The run consisted of four chains, one of which was the ‘cold’ chain and three of which were heated according to the default heating method parameters of MrBayes (Ronquist & Huelsenbeck 2003). Each chain was run for nine million generations initiated from a random starting tree and sampled every 500 generations for a total of 18,001 tree samples per run. After nine million runs, the standard deviation of split frequencies between simultaneous runs was less than 0.01. Sampled genealogies from each run were combined in Logcombiner (Rambaut & Drummond 2015) for a total of 36,002 sampled genealogies. Tree Annotator (Rambaut & Drummond 2013) was used to summarize the trees into a single maximum clade credibility tree. The dendrogram was visualized in FigTree v.1.3.1 (Rambaut 2009).
